# CRISPR/Cas-Based Prenatal Screening for Aneuploidy: Challenges and Opportunities for Early Diagnosis

**DOI:** 10.3390/medicina61040610

**Published:** 2025-03-27

**Authors:** Irisappan Ganesh, Ilangovan Karthiga, Manoranjani Murugan, Kumar Rangarajalu, Vishnu Bhat Ballambattu, Sambandam Ravikumar

**Affiliations:** 1 Department of Medical Biotechnology, Aarupadai Veedu Medical College & Hospital, Vinayaka Mission’s Research Foundation (Deemed to be University), Puducherry 607402, India; ganesh.irisappan@avmc.edu.in (I.G.); manoranjanimurugan@avmc.edu.in (M.M.); 2 Department of Biochemistry, Aarupadai Veedu Medical College & Hospital, Vinayaka Mission’s Research Foundation (Deemed to be University), Puducherry 607402, India; karthigasaiba@gmail.com (I.K.); kumar.rangarajalu@avmc.edu.in (K.R.); 3 Advisor—Medical Research & Publications, Aarupadai Veedu Medical College & Hospital, Vinayaka Mission’s Research Foundation (Deemed to be University), Puducherry 607402, India; drvishnubhat@yahoo.com

**Keywords:** prenatal screening, aneuploidy, CRISPR/Cas, cell-free fetal DNA, molecular diagnostics

## Abstract

Aneuploidy is increasingly recognized globally as a common cause of miscarriage among expectant mothers. The existing prenatal screening techniques for detecting aneuploidy have several limitations. The ability to diagnose aneuploidy early in a non-invasive manner is not feasible with the current screening methods, as they may produce false positive or false negative results. Recently, the widely used gene editing tool CRISPR/Cas has shown great promise in diagnostics. This review summarizes the prenatal screening tests used in the first trimester to assess aneuploidy conditions. Additionally, we discuss the advantages and disadvantages of molecular diagnostic tests, including the benefits and challenges of CRISPR/Cas-based trisomy detection. Thus, the proposed prenatal screening using CRISPR/Cas could provide significant benefits to expectant mothers by potentially enabling the early diagnosis of trisomy, helping to prevent miscarriage and birth defects. Furthermore, it opens new avenues for research, allowing clinicians and researchers to develop, optimize, and implement CRISPR/Cas-based prenatal screening assays in the future.

## 1. Introduction

Aneuploidy is a common genetic abnormality linked to several diseases, including cancer, infertility, and birth abnormalities. It is a condition characterized by an abnormal number of chromosomes, such as the presence of additional chromosomes or the absence of certain chromosomes, including duplications, translocations, and deletions [1]. Chromosomal abnormalities affect 1 in 150 live births, and the risk of aneuploidy increases with maternal age. Nonviable pregnancies, birth abnormalities, or infant mortality can result from the insertion or deletion of chromosomal regions, as each chromosome contains hundreds of genes [2].

Aneuploidy can occur in any cell type in the body, including somatic and germ cells. It arises from several different mechanisms. A common mechanism is a nondisjunction, which happens when chromosomes fail to separate properly during meiosis, leading to gametes with an abnormal number of chromosomes. Nondisjunction can occur during meiosis I or meiosis II, resulting in either monosomy or trisomy [3]. Monosomy refers to the loss of one chromosome, while trisomy refers to the gain of one chromosome. Additionally, aneuploidy can be caused by errors in mitosis, the process of cell division that occurs in somatic cells. Another mechanism that can cause aneuploidy is chromosomal translocation, which occurs when parts of two different chromosomes exchange places. This can result in gametes with extra or missing genetic material and is often associated with cancer [4].

The most prevalent chromosomal disorder that affects 1 in 800 live births is Down syndrome (Trisomy 21) [5]. Every year, approximately 6000 infants in the United States are affected, with 95% of cases due to nondisjunction involving chromosome 21 [6]. Other common examples of aneuploidy include Turner syndrome, caused by a missing X chromosome in females, and Klinefelter syndrome, caused by an extra X chromosome in males [7].

This review examines first-trimester prenatal screening methods for identifying aneuploidy in pregnant women, highlighting the benefits and limitations of molecular diagnostic tests available during this period. It also explores the potential of the CRISPR-Cas technique for diagnosing trisomy, detailing its advantages and challenges.

## 2. Sampling

### 2.1. Chorionic Villus Sampling

Chorionic villus sampling (CVS) is a medical procedure employed for the purpose of genetic diagnosis, typically performed between the 10th and 13th weeks of gestation. This technique enables the collection of placental tissue for subsequent analysis. The two primary methodologies for conducting CVS are transabdominal and transcervical approaches. Although transcervical CVS is associated with an increased incidence of spontaneous pregnancy loss, it is frequently the preferred method when the placenta is positioned posteriorly or when the positioning of the bowel obstructs the transabdominal approach. The principal advantage of CVS lies in its capacity to provide early and definitive chromosomal analysis. However, it is important to note that this procedure is invasive and carries a risk of pregnancy loss, which ranges from 0.6% to 4.6% [8].

### 2.2. Amniocentesis

Amniocentesis (AMC) is conducted using ultrasound guidance between 16 and 28 weeks of pregnancy, during which around 20 mL of amniotic fluid is extracted. The abortion rate associated with this procedure is 1%, but it rises significantly if conducted before 16 weeks [9]. A clinical study highlighted notable distinctions in total fetal loss (7.6% vs. 5.9%) and amniotic fluid leakage (3–5% vs. 1.5%) when comparing the early AMC group (at 13 weeks) to the mid-AMC group (after 15 weeks) [10].

### 2.3. Cell-Free Fetal DNA

During pregnancy, villous trophoblasts continually turnover, releasing apoptotic material into the maternal circulation. This includes cell-free fetal DNA (cffDNA), commonly referred to as “fetal DNA”, which originates from the placenta. CffDNA screening is a non-invasive prenatal testing method used to analyze short fragments of fetal DNA in maternal serum samples during gestation. The concentration of fetal DNA in maternal blood increases with gestational age, surpassing 10% by the 10th week of gestation and progressively rising throughout pregnancy to levels of 20–30% during the third trimester [11]. The quantity of isolated cffDNA can be determined by measuring Y-chromosome fractions and the amount of variably methylated regions in both maternal and fetal DNA [12]. In 2006, Chan et al. reported that the promoter region of the RASSF1A gene is hypomethylated in maternal blood cells and hypermethylated in fetal cells [13]. cffDNA molecules are fragmented and typically display two peaks of approximately equal height at 143 and 166 bases. Generally, cffDNA fragments are shorter than 300 bases, while fragments of maternal cell-free DNA exceed 300 bases. Consequently, cffDNA can be enriched by collecting maternal plasma cell-free DNA fragments that are under 300 bp in length [14]. Although electrophoresis has been utilized to differentiate short cffDNA fragments from larger maternal-derived cell-free DNA fragments, its accuracy remains limited. Recent research has explored alternative techniques, such as microfluidics and silica particles, to improve the separation of cffDNA from maternal cfDNA [15].

## 3. Prenatal Screening Tests

Throughout all trimesters of pregnancy, women have access to screening tests for detecting aneuploidy. In the first trimester, options such as triple, quad, and penta screens, along with ultrasonographic examinations, are available in a combined screening test. Integrated, sequential, and contingent screening tests, however, are restricted to the second and third trimesters of gestation [5].

Screening tests conducted between 10 and 13 weeks of gestation constitute the first-trimester screening, which combines serum screening markers such as free beta human chorionic gonadotrophin and pregnancy-associated plasma protein A with an ultrasound assessment of nuchal translucency. An NT measurement exceeding 3.0 mm indicates a risk of aneuploidy and potential structural malformations [5]. Although these screening tests can be conducted early in pregnancy, it is crucial that certified and skilled sonographers perform the nuchal translucency measurements, as even a 0.5 mm variation can significantly impact the test’s sensitivity. The detection sensitivity of NT improves only after the 11-week mark in gestation [16,17]. In recent years, first-trimester screening tests have been refined for the early diagnosis of fetal aneuploidy. Additionally, the previous recommendation of using 35 years of age as a threshold for invasive prenatal diagnostic testing is no longer valid. This updated guideline replaces the American College of Medical Genetics (ACMG) policy [18].

### 3.1. Triple, Quad, and Penta Screening

The quadruple marker screen, also known as the quad screen, is a screening procedure performed between 15 and 22 weeks of pregnancy. It measures dimeric inhibin A, alpha-fetoprotein (AFP), hCG, and unconjugated estriol while considering the patient’s age, race, weight, gestational age, gestational diabetes status, and the number of fetuses. Besides screening for aneuploidy, this technique can also diagnose open neural tube defects. Additionally, it does not require specialized sonographers to carry out the screening. The detection rate is 81%, with a positive rate of 5%, which is more modest compared to first-trimester screening [5]. Providing inaccurate gestational dates and details during blood sampling may reduce the accuracy of the screening. Variations in the quad screen include the Penta screen, which measures hyperglycosylated hCG, along with the quad screen markers [19]. Another variation, known as the triple screen, measures serum AFP, hCG, and unconjugated estriol [20]. However, both screening tests are not widely used as they need to improve their test characteristics.

### 3.2. Ultrasonographic Screening

Ultrasonographic screening is routinely conducted for all pregnant women throughout their pregnancy. This procedure is carried out to confirm the due date and to assess potential birth defects [21]. It helps identify significant structural abnormalities, including cardiac anomalies and minor ultrasound soft markers for aneuploidy. First-trimester ultrasound is performed using transvaginal and transabdominal methods to evaluate major anomalies [21]. Second-trimester abdominal ultrasound is conducted to assess for malformations or markers linked to aneuploidy.

### 3.3. Non-Invasive Prenatal Screening (NIPT)

Non-invasive prenatal testing (NIPT) is currently promoted as a screening tool for fetal trisomies 21, 13, and 18. Certain companies additionally broaden their offerings to encompass sex chromosomal aneuploidy (SCA), various other autosomal aneuploidies, along with microduplication and microdeletion syndromes, all of which can be technically identified through NIPT [22]. Cell-free DNA that engages more than 4% in the maternal sample is ideal for screening aneuploidy through sequencing techniques [23]. Among the various screening methods, cell-free DNA screening is a non-invasive prenatal approach that provides a 99% detection rate for trisomy 21. However, compared to trisomy 21, the detection rates for other trisomies, such as trisomy 18, 13, and sex chromosomal anomalies, are lower [24]. Additionally, cell-free DNA screening can be utilized to check for maternal chromosomal abnormalities, such as maternal mosaicism, as well as infrequently occurring maternal cancers [25]. Most early non-invasive prenatal testing relied on various polymerase chain reaction (PCR) and microarray techniques to identify chromosomal aberrations in fetal DNA derived from maternal plasma [26]. This type of screening may yield false positive results, particularly in cases of vanishing twins or confined placental mosaicism. A common reason for failing to obtain a result is a low fetal DNA fraction in maternal plasma. Many laboratories use a fetal fraction cut-off of 4% as the minimum for test interpretation. Factors that seem to correlate with fetal fraction include maternal weight, gestational age, and serum markers PAPPA and hCG [27]. Although the detection rate for trisomy 21 has improved with cell-free DNA screening, other chromosomal abnormalities not evaluated by this method can only be identified through serum screening approaches [28]. This can delay the diagnosis of fetal abnormalities caused by trisomies, potentially leading to birth defects and miscarriages. Therefore, there is an urgent need to discover a diagnostic method to detect trisomies quickly and effectively using cell-free fetal DNA.

## 4. Molecular Diagnostic Methods

**Figure 1:**
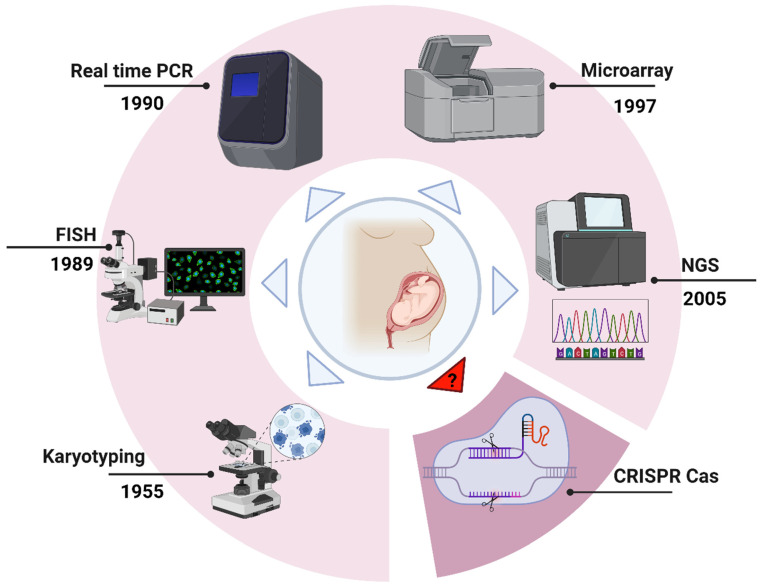
Various techniques are used in prenatal screening to detect aneuploidy. **1.** Karyotyping **2.** Fluorescent in situ hybridization (FISH) **3.** Real-time quantitative polymerase chain reaction (RT-qPCR) **4.** Microarray **5.** Next generation sequencing (NGS) and **6.** A proposed advanced technique using CRISPR/Cas system.

The diagnostic methods conducted during the first trimester encompass karyotyping, fluorescence in situ hybridization (FISH), real-time quantitative polymerase chain reaction (qPCR), microarray analysis, and gene sequencing, as illustrated in Figure 1.

### 4.1. Karyotyping

Prenatal screening through karyotyping is a method for analyzing the chromosomes of a developing fetus during pregnancy. Karyotyping is considered the “gold standard” technique for diagnosing fetal aneuploidies. This method involves examining a sample of fetal cells obtained through amniocentesis or chorionic villus sampling (CVS) for abnormalities in chromosome number or structure [29,30,31]. While karyotyping has been widely utilized for several decades, it has several drawbacks, such as invasive sampling procedures, limited detection capabilities since it can only identify chromosomal abnormalities related to the number or structure of chromosomes, a long turnaround time, the potential for false positive results due to technical errors or culture contamination, the necessity for skilled personnel to perform karyotyping, and various ethical concerns [32]. These limitations have prompted the search for alternative prenatal screening methods.

### 4.2. Fluorescent In Situ Hybridization (FISH)

FISH (Fluorescence In Situ Hybridization) represents a form of prenatal screening that employs fluorescent probes to identify specific genetic abnormalities in fetal cells obtained through amniocentesis or chorionic villus sampling (CVS) [21]. This method can be utilized to detect chromosomal abnormalities linked to conditions such as Down syndrome, Turner syndrome, and certain types of leukemia [33,34,35]. FISH offers the advantage of delivering rapid results and can be employed in conjunction with other genetic testing techniques, such as karyotyping, to perform a comprehensive examination of the fetus’s genetic profile [36]. Nonetheless, FISH is subject to certain limitations, including a restricted ability to identify abnormalities involving small or large chromosomal regions, as the fluorescent signals may either be undetectable or challenging to interpret [37]. Furthermore, invasive procedures such as amniocentesis and CVS, which are prerequisites for conducting FISH, carry risks of miscarriage and other complications [38]. Lastly, similar to other prenatal screening tests, the results of FISH are associated with the possibility of false positives and false negatives, which necessitate verification through additional testing [36].

### 4.3. Real-Time Quantitative PCR (RT-qPCR)

Real-time quantitative PCR (RT-qPCR) is a molecular technique widely used for detecting and quantifying nucleic acid sequences [39]. RT-qPCR helps identify fetal chromosomal abnormalities, such as Patau syndrome (trisomy 13), Edwards syndrome (trisomy 18), and Down syndrome (trisomy 21) [40,41]. During prenatal screening, fetal DNA isolated from maternal blood is amplified and quantified using RT-qPCR, which can detect low levels of DNA [42]. RT-qPCR-based prenatal screening offers advantages like non-invasive procedures, rapid detection, and high sensitivity and specificity, eliminating the need for post-PCR processing steps [43]. However, the limited amount of cell-free fetal DNA present during the early gestational period makes it challenging to screen for fetal abnormalities [44]. In quantitative fluorescence PCR (QF-PCR) studies, a thorough investigation reveals that primer mismatching can disrupt the original ratio during the amplification process, leading to the incomplete binding of primers to the target sequence [45]. Although QF-PCR is highly precise and reliable, a significant drawback of this technique is the applicability of informative polymorphisms observed in the human population [46,47]. These issues may result in false negatives that could obscure the severity of fetal abnormalities.

### 4.4. Microarray

Microarray-based prenatal screening methods detect chromosomal abnormalities in fetal samples using a microchip. They provide high accuracy in identifying chromosomal abnormalities and have a lower rate of false positives compared to other screening techniques like karyotyping, FISH, and RT-qPCR. This method can identify a broader range of genetic abnormalities than FISH, including microdeletions and microduplications [48]. Additionally, microarray screening is non-invasive and carries no risk of miscarriage [49]. However, its limitations include the restricted ability to determine the severity of certain genetic disorders, such as low-level mosaicism caused by aneuploidy. Consequently, this can lead to false positives and false negatives, potentially resulting in missed diagnoses. Microarray testing cannot detect molecularly balanced chromosomal rearrangements [35]. Furthermore, compared to other prenatal screening tests, microarray is expensive and requires technical expertise to administer, which may limit its availability and accessibility in some healthcare settings [50]. Additionally, the use of microarray raises ethical concerns, such as the potential for uncovering sensitive genetic information [51].

### 4.5. Gene Sequencing

Gene sequencing is an effective prenatal screening technique that involves extracting DNA from fetal cells found in amniotic fluid, placenta, or maternal blood. The two main types of prenatal screening techniques are whole genome sequencing (WGS) and targeted gene sequencing, which focus on the whole genome and/or specific genes or gene mutations [52]. WGS offers more accurate results but is expensive and time-consuming [53], while targeted gene sequencing is faster and less costly but only detects specific genes or gene mutations [54]. The advantages of sequencing include early detection, high accuracy, comprehensive screening, and non-invasive sampling. However, like other screening methods, it has limitations, such as the potential for false positive results, limited information on genetic disorders, the cost of kits, and ethical concerns, which can make it a less feasible option for prenatal screening [55].

In addition to the aforementioned technologies, MLPA (Multiplex Ligation-dependent Probe Amplification) is used for prenatal genetic testing [55]. Although these technologies are effective in diagnosing fetal aneuploidies, they require trained professionals, can yield false positive results, and entail high costs that limit their use in routine pregnancy screening. Therefore, the primary challenge is developing a low-cost, rapid, selective, sensitive, and efficient technique for effectively detecting aneuploidies.

Aneuploidy is responsible for 86% of the miscarriages occurring during the first trimester [56]. Detecting aneuploidy through visual or symptom-based methods is not feasible during pregnancy, which is essential to avoid fetal loss or the risk of birth abnormalities. Therefore, a rapid and accurate screening method for aneuploidy is vital for effective management. In molecular diagnostics, nucleic acids are the ideal biomarkers, identified using qRT-PCR, FISH, Microarray, and gene sequencing techniques. However, these methods require advanced equipment, costly kits, and trained personnel. Consequently, there is an immediate demand for a fast, highly sensitive, and precise diagnostic tool or kit for the early detection of chromosomal aneuploidy.

### 4.6. CRISPR/Cas Based Diagnostics

CRISPR/Cas systems are essential components of the prokaryotic adaptive immune system, cleaving foreign nucleic acids with RNA-guided nucleases [57]. Cas utilizes short RNA molecules as templates to make highly sequence-specific cuts in DNA, which can be harnessed to insert genes or modify nucleotide sequences precisely [58]. CRISPR/Cas represents third-generation gene editing technology, following transcription activator-like effector nucleases (TALENs) and zinc finger nucleases (ZFNs) [59]. Previously, CRISPR/Cas was primarily used for purposes such as genome editing [60], epigenome editing [61], transcriptome analysis [62], bioimaging of nucleic acids [63], and recording cellular events [64]. Recently, it has gained prominence in nucleic acid detection, particularly with the discovery of SHERLOCK [65] and DETECTR [66], which streamlined sample preparation methods, reduced PCR analysis operation time, and lowered chemical expenses in diagnostics, enhancing its usability. Additionally, the precise identification of phytopathogens in point-of-care (POC) disease diagnostics was achieved using CRISPR in combination with isothermal amplification and lateral flow assay (LFA), all without the need for sophisticated instruments or specialized technical skills [67,68].

The CRISPR-Cas systems have largely been categorized into two classes: class 1 systems, including types I, III, and IV, which utilize multiprotein complexes to degrade foreign nucleic acids, and class 2 systems, comprising types II, V, and VI, which employ single proteins [69]. A breakthrough in nucleic acid detection has been the discovery of Cas13a (formerly C2c2) and Cas12a (formerly Cpf1), both of which exhibit collateral cleavage activity [70] (Table 1). The PAM for Cas12a, typically 5′-TTTV-3′, is positioned upstream of the target DNA. Once it identifies the PAM, the target DNA is unwound and hybridized with RNA [71]. Cas12a contains a single nuclease site, resulting in DNA strands being cleaved at this same nuclease site, leading to a staggered double-strand break. In addition to catalyzing site-specific cleavage activity on target dsDNA/ssDNA, Cas12a/Cas12b-crRNA can also initiate non-specific ssDNA cleavage activity once it binds to target dsDNA/ssDNA [72].

### 4.7. CRISPR/Cas-Based Diagnostics of Infectious Disease

As a global health issue, infectious diseases impact billions of people around the world. The development of rapid and sensitive diagnostic tools is essential for effectively managing patients and curtailing the spread of disease [73]. The CRISPR-Cas system has recently gained considerable traction as a method for detecting infectious diseases, including SARS-CoV-2, tuberculosis, dengue, monkeypox virus, etc., (Table 2). Viral infections are the most commonly investigated area for CRISPR-based diagnostic systems, where Cas12a type V enzymes can directly bind to DNA targets in a three-stage process in which guide RNA directs the Cas12a enzyme to the specific double-stranded DNA sequence in the viral genome [70]. The Cas12a enzyme indiscriminately cleaves single-stranded DNA molecules that are bound to quencher molecules and reporter fluorophores, resulting in the emission of a fluorescent signal [73].

Previously, Kang et al. developed the nucleic acid lateral flow immunoassay (NALFIA), a novel method that employs recombinase polymerase amplification (RPA) and Cas12a for the rapid and accurate detection of plant pathogens, specifically *Magnaportheoryzae Triticum* (MoT). This method requires just 30 min for pathogen detection, eliminating the need for complicated laboratory instruments or skilled technicians [67]. In a recent study, Ali et al. introduced Bio-SCAN (Biotin-coupled CRISPR-based nucleic acid detection) to detect SARS-CoV-2. This method combines reverse transcription-RPA (RT-RPA), recombinant biotin-labeled nuclease-dead Cas9 (bio-dCas9), and lateral flow assays (LFA) to achieve 100% specificity in SARS-CoV-2 diagnosis [93]. In another recent study, the rapid detection of phytopathogens, transgenes, and herbicide-resistant alleles was accomplished by optimizing Bio-SCAN for the point-of-care testing (POCT) platform [68].

The capability to detect pathogens and to identify the specific gene or chromosomal location by precisely adjusting the gRNA to target distinct nucleic acid fragments within the specified region represents one of the most notable features of CRISPR-based detection technologies [94], and the detection process only requires ~1 h. Furthermore, the use of RPA to enhance the target region of the gene prior to detection eliminates the need for specialized instruments like PCR for amplification. Consequently, CRISPR-based detection technology requires minimal reagents, selectively targets the area of interest, and reduces detection time, which is urgently needed for developing and low-income countries. As a result, CRISPR-based technologies can serve as point-of-care nucleic acid detection systems and deployable diagnostic tools capable of identifying and mitigating outbreaks and emerging epidemics.

### 4.8. CRISPR/Cas-Based Diagnostics of Non-Infectious Disease

The CRISPR/Cas9/Cas12 system is recognized as a valuable tool for identifying oncogenes and other mediators of cancer, and it has been integrated into cancer research [70]. Recently, the CRISPR-Cas system has been widely employed to detect non-infectious diseases such as cancer and congenital erythropoietic porphyria, etc., (Table 3). This technology has the potential to enhance the detection of non-infectious diseases by identifying specific genetic mutations or biomarkers associated with these conditions [95]. This capability enables early diagnosis and the development of personalized treatment strategies, especially in cancer, where identifying specific genetic alterations is crucial [95]. This process involves designing guide RNAs that specifically target relevant DNA sequences, allowing the CRISPR-Cas complex to bind to and indicate the presence of disease-related genetic markers, often using detectable signals like fluorescence.

### 4.9. CRISPR/Cas-Based Diagnostics for Trisomy Detection

CRISPR-Cas is a promising genome editing technology (Table 4) that can be widely utilized in diagnostics [101]. The CRISPR-Cas9 tool has the potential to generate indels in chromosomes, with its DNA-editing capability stemming from the wild-type Cas9 protein’s ability to create double-stranded breaks at the target site defined by the custom-designed sgRNA [102]. During the repair of DNA breaks, nonhomologous end joining (NHEJ) machinery often produces indels; however, homology-directed repair (HDR) machinery can utilize the complementary template, achieving a more precise gene editing process.

The genetic disorder for which CRISPR-Cas-based diagnostics show promising potential is the detection of trisomy, a common genetic anomaly linked to various health issues, including birth defects and other aneuploidies [108]. These diagnostics for trisomy depend on identifying DNA sequences that are unique to specific chromosomes. The CRISPR-Cas guide RNAs can be designed using specific tools like Benchling or Chop-Chop. Generally, when targeting a specific sequence, the change in fluorescence or any other detectable signal can be measured using a spectrofluorometer or plate reader. One of the major advantages of CRISPR-Cas-based diagnostics is their high specificity and the capacity to detect fewer copies of DNA at an early stage [109].

The CRISPR-Cas-based diagnostics for trisomy will function like FISH probes that target specific chromosomal regions [34]. The guide RNA is designed to bind to DNA sequences unique to the targeted chromosomal region and is labeled with a fluorescent tag that emits a signal when excited by a specific wavelength of light [34]. Measuring the fluorescence intensity of the labeled probe determines the existence of an abnormal number of chromosomes. Another example of CRISPR-Cas-based diagnostics for trisomy is the use of an RT-qPCR-based assay that targets a specific chromosomal region [110]. The assay probe or primer can be designed to amplify specific DNA sequences unique to the targeted chromosomal region and is labeled with a fluorescent tag that emits a signal when excited by a specific wavelength of light. Measuring the fluorescence intensity of the labeled probe will determine the abnormal number of chromosomes in trisomy [111].

### 4.10. Advantages of CRISPR/Cas-Based Diagnostics for Trisomy

There are several advantages to using CRISPR-Cas-based diagnostics for detecting trisomy. One primary advantage is the high specificity and early detection of trisomies. This enables earlier identification and intervention, which can lead to improved outcomes for individuals with trisomy. Moreover, these diagnostics are highly sensitive and can identify trisomy even when only a few cells have an abnormal number of chromosomes. This sensitivity is particularly crucial for prenatal screening, where detecting aneuploidy in a small number of cells can signify a high risk of birth defects or other complications [112].

**Figure 2:**
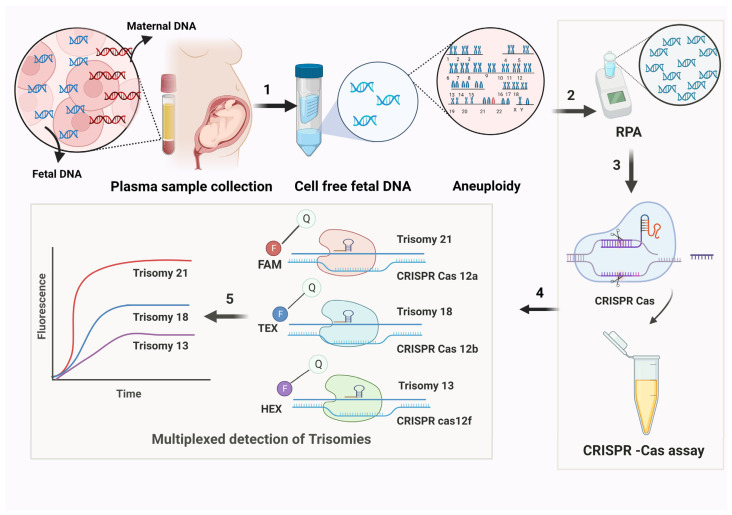
Proposed non-invasive prenatal (NIPT) screening by a clustered regularly interspaced short palindromic repeats (CRISPR)/Cas-based assay. **1.** Maternal blood samples will be collected from the gestational mother and processed to isolate the cell-free fetal DNA (cffDNA) using the kit method. **2.** The cffDNA will be amplified using the fluorophore-labeled primers designed for recombinase polymerase amplification (RPA). **3.** The RPA mixture will contain single guide (sg) RNA and Cas nucleases designed to target the chromosomal regions to detect aneuploidies by CRISPR/Cas assay. **4.** For multiplex detection, various sgRNAs will be programmed to target trisomies such as Trisomy 13, Trisomy 18, and Trisomy 21. The detection of 3 targets will be favored by adding reporters for Cas12f, Cas12b, and Cas12a in HEX (hexachlorofluorescein), TEX (TEX615), and FAM channels. The sgRNAs will recognize and bind to the fluorophore-targeted cffDNA resulting in the formation of the fluorophore-DNA-Cas-sgRNA complex. This will result in the generation of fluorescence by the separation of fluorophore and quencher. **5.** The fluorescence generated will be measured using a spectrofluorometer or plate reader with distinct absorbance and emission wavelengths.

This approach allows pregnant women to be screened for aneuploidies like trisomy 21, 18, and 13 through a blood test, facilitating the extraction of fetal DNA from maternal plasma cells rather than traditional amniocentesis. Additionally, CRISPR-Cas-based diagnostics for trisomy offer a relatively straightforward, quick, and cost-effective platform that can be implemented in a variety of laboratory environments (Figure 2). This accessibility will enable more researchers and clinicians to utilize it, enhancing the availability and affordability of trisomy screening.

### 4.11. Advantages of CRISPR/Cas-Based Diagnostics in ARF and IVF Conception

CRISPR-Cas-based diagnostics have the capacity to screen embryos obtained through assisted reproductive techniques (ART) and in vitro fertilization (IVF), given the potential risk of trisomy 18 or 21 [113,114]. Since trisomy is associated with intellectual disabilities [115], this technology can be employed to evaluate and mitigate the implications of ART, particularly in relation to neuro-psycho-motor complications [116]. Moreover, this technology could have a profound impact on complete or partial ectogenesis by enabling the screening of embryos created within an artificial uterus. However, this will raise the ethical issues which need to be considered with caution and responsibility [117].

### 4.12. Challenges of CRISPR/Cas-Based Diagnostics for Trisomy

Despite the various applications of CRISPR-Cas-based diagnostics for trisomy, several challenges remain to be addressed. A primary challenge involves the development of reliable and accurate guide RNAs or assays that target specific chromosomal regions. This requires a comprehensive understanding of genomic areas unique to each chromosome, along with the potential for cross-reactivity with other chromosomal regions. Additionally, optimizing the CRISPR-Cas system for its application in diagnostics presents another hurdle. Although the Cas protein demonstrates high specificity, it possesses the potential to induce off-target effects through binding to non-target genomic regions [118]. Such interactions can result in false positives or false negatives in diagnostic assays, thereby complicating the accuracy of trisomy screening. To mitigate this issue, researchers are exploring new Cas variants that exhibit heightened specificity and reduced off-target effects. For instance, the application of high-fidelity Cas enzymes has been shown to diminish off-target effects by as much as 50-fold when compared to wild-type Cas [119]. Furthermore, the design of sgRNAs utilizing specialized bioinformatics tools is essential in minimizing the impact of off-target CRISPR-Cas complexes. When cleavage sites are typically located within predicted target sequences rich in uracil, ssRNA loops, or ssRNA junctions with dsRNA, folding prediction software may be employed. Additional strategies include the use of RNA-guided FokI nucleases, which provide enhanced specificity and fewer off-target effects in comparison to Cas [120]. Another significant concern in CRISPR-Cas-based diagnostics for trisomy is the stringent validation and standardization of diagnostic assays to ensure specificity, sensitivity, and reliability. This necessitates the implementation of appropriate controls, statistical analysis, and comparisons with gold-standard methods.

A prevalent ethical consideration pertains to the safety of utilizing CRISPR-Cas-based technologies. In contrast to therapeutic applications, the diagnostic implementations of CRISPR generally exhibit non-invasive characteristics and can be conducted under in vitro conditions for prenatal diagnosis [121]. Consequently, CRISPR/Cas may represent a promising technological advancement in the realm of prenatal diagnosis.

## 5. Conclusions

Aneuploidy is a condition characterized by cells containing an atypical number of chromosomes. The current prenatal screening techniques and methods have several limitations in diagnosing aneuploidy during the early gestational period. The CRISPR-Cas-based system, primarily used to detect various infectious and non-infectious diseases, may have the potential to identify aneuploidy in early gestation. As this technology is widely utilized for editing and detecting microdeletions, it can be leveraged for diagnostics that contribute to the qualitative or quantitative detection of chromosomal aberrations. This approach may reduce the need for invasive diagnostic procedures such as amniocentesis and bone marrow biopsy. Furthermore, this technology can be applied in oncology diagnostics to identify chromosomal translocations and copy number variations in cancer patients, facilitating personalized therapies. However, challenges such as appropriate guide RNA design, off-target effects, and developing effective strategies limit its use. Therefore, employing CRISPR-Cas technology for diagnostic applications may enable healthcare professionals to recognize its capabilities without encountering the associated challenges.

## Figures and Tables

**Table 1:** Characteristics of various CRISPR/Cas systems.

Protein Family	Cas9	Cas12	Cas13	Cas14
Bacteria	*Streptococcus pyogenes*	*Lachnospiraceae*	*Leptotrichia shahii*	DPANN (Archea)
Nuclease domine	RUVC&HNH	RUVC	RUVC	RUVC
Guide RNA length	17–150 nt	41–44 nt	50–64 nt	−140 nt
PAM Sequence	NGG	TTTN	Protospacer flanking site	TTTA
Cleavage site	3–4 nt upstream of PAM	−18 bases 3 of PAM	3′ end of the target RNA	3′ end of the target region
Target nucleic acid	DNA	DNA	RNA	DNA
Termini of cleavage	Blunt end	Overhang ends	Sticky end	Sticky end

This table compares Cas9, Cas12, Cas13, and Cas14 proteins alongside their bacterial origins, nuclease domain, guide RNA size, PAM sequences, cleavage sites, target nucleic acids, and cleavage termini.

**Table 2:** List of CRISPR/Cas 12a nucleases in the detection of infectious diseases.

S.No	Type of Cas	Title	Infectious Disease	Reference
1	Cas12a	A new method for the detection of Mycobacterium tuberculosis based on the CRISPR/Cas system	Tuberculosis	Zhang et al. [74]
2	Cas12a	Clustered regularly interspaced short palindromic Repeat/Cas12a mediated multiplexable and portable detection platform for GII genotype Porcine Epidemic Diarrhoea Virus Rapid diagnosis	Genotype Porcine Epidemic Diarrhea Virus	Qian et al. [75]
3	Cas12a	Clinical Validation of Two Recombinase-Based Isothermal Amplification Assays (RPA/RAA) for the Rapid Detection of African Swine Fever Virus	African Swine Fever Virus	Fan et al. [76]
4	Cas12a	CRISPR-Cas12-based detection of SARS-CoV-2	COVID-19	Beroughton et al. [77]
5	Cas12a	Proximity sequence enhanced CRISPR-Cas12a connected through hybridization chain reaction for sensitive biosensing of dengue virus	Dengue	Zhong et al. [78]
6	Cas12a	Target nucleic acid amplification-free detection of *Escherichia coli* O157:H7 by CRISPR/Cas12a and hybridization chain reaction based on an evanescent wave fluorescence biosensor	*E. coli*	Song et al. [79]
7	Cas12a	A ratiometric fluorescent biosensor for rapid detection of *Burkholderia pseudomallei* by dual CRISPR/Cas12a trans-cleavage assisted signal enhancement	*Burkholderia pseudomallei*	Lin et al. [80]
8	Cas12a	Rapid and sensitive detection of *Pseudomonas aeruginosa* by isothermal amplification combined with Cas12a-mediated detection	*Pseudomonas aeruginosa*	Huang et al. [81]
9	Cas12a	Violet phosphorene nanosheets coupled with CRISPR/Cas12a in a biosensor with a low background signal for onsite detection of tigecycline-resistant hypervirulent *Klebsiella pneumoniae*	*Klebsiella pneumoniae*	Li et al. [82]
10	Cas12a	Isothermal Amplification and CRISPR/Cas12a-System-Based Assay for Rapid, Sensitive and Visual Detection of *Staphylococcus aureus*	*Staphylococcus aureus*	Xu et al. [83]
11	Cas12a	A novel CRISPR/Cas12a biosensor for sensitive detection of *Helicobacter pylori* from clinical patients	*Helicobacter pylori*	Yu et al. [84]
12	Cas 12a	Naked-eye on-site detection platform for *Pasteurella multocida* based on the CRISPR-Cas12a system coupled with recombinase polymerase amplification	*Pasteurella multocida*	Hao et al. [85]
13	Cas 12a	CRISPR/Cas12a-Based Detection Platform for Early and Rapid Diagnosis of Scrub Typhus	*Scrub Typhus*	Bharadwaj et al. [86]
14	Cas 12a	Rapid and Sensitive Detection of *Vibrio vulnificus* Using CRISPR/Cas12a Combined with a Recombinase-Aided Amplification Assay	*Vibrio vulnificus*	Xiao et al. [87]
15	Cas 12a	The combination of RPA-CRISPR/Cas12a and Leptospira IgM RDT enhances the early detection of leptospirosis	*Leptospirosis*	Jirawannaporen et al. [88]
16	Cas 12a	CRISPR-Cas12a assisted specific detection of mpox virus	Mpox virus	Singh et al. [89]
17	Cas 12a	CRISPR-Cas12a-Mediated Hue-Recognition Lateral Flow Assay for Point-of-Need Detection of *Salmonella*	*Salmonella*	Yuan et al. [69]
18	Cas 12a	Rapid detection of monkeypox virus using a CRISPR Cas12a mediated assay: a laboratory validation and evaluation study	Monkeypox virus	Low et al. [90]
19	Cas 12a	Point-of-care detection of *Neisseria gonorrhoeae* based on RPA-CRISPR/Cas12a	*Neisseria gonorrhoeae*	Tu et al. [91]
20	Cas 12a	Rapid detection of avian influenza virus based on CRISPR-Cas12a	Avian influenza virus	Zhou et al. [92]

This table summarizes various studies utilizing Cas12a for the detection of infectious diseases, listing the pathogen detected and detection method.

**Table 3:** List of CRISPR/Cas12a nucleases in the detection of non-infectious diseases.

S.No	Type of Cas	Title	Genes	Reference
1	Cas12a	Cas12a-based one-pot SNP detection with high accuracy	*CYP2C19*	Zhang et al. [96]
2	Cas12a	In Vitro CRISPR-Cas12a-Based Detection of Cancer-Associated *TP53* Hotspot Mutations Beyond the crRNA Seed Region	Cancer-Associated *TP53* Hotspot	Kohabir et al. [97]
3	Cas12a	Detecting Melanocortin 1 Receptor Gene’s SNPs by CRISPR/enAsCas12a	Melanocortin 1	Yang et al. [98]
4	Cas12a	Rapid detection of isocitrate dehydrogenase 1 mutation status in glioma based on Crispr-Cas12a	Isocitrate dehydrogenase	Feng et al. [99]
5	Cas12a	Rapid and ultra-sensitive early detection of cervical cancer using CRISPR/Cas12-based assay based on methylated SEPT9	SEPT9	Xu et al. [100]

This table captures the usage of Cas12a for identifying mutation of the genes related to non-infectious diseases like cancer and other disorders.

**Table 4:** List of CRISPR-Cas9 and CRISPR-Cas12a nucleases in editing and diagnostics of prenatal screening.

S.No	Types of Cas	Title	Chromosome /Locus	Reference
1	Cas12a	Development of the Cas12a-based microdeletion and micro insertion detection system	Pde6b-KO mice and Grin3A-KO mice	Chirinskaite et al. [103]
2	Cas9	CRISPR/Cas9-mediated targeted chromosome elimination	TKNEO, XIST Transgene	Zuo et al. [104]
3	Cas9	CRISPR-Cas9 genome editing induces megabase-scale chromosomal truncations	*UROS* locus to model and correct congenital erythropoietic porphyria	Cullot et al. [105]
4	Cas12a	Engineering microdeletions and microduplications by targeting segmental duplications with CRISPR	16p11.2 and 15q13.3	Tai et al. [106]
5	Cas12a	Mitigation of chromosome loss in clinical CRISPR-Cas9-engineered T cells	TRAC locus	Tsuchida et al. [107]

This table emphasizes the application of Cas9 and Cas12a in detecting chromosomal deletions, duplications, and mutations during prenatal genetic screening.

## Data Availability

The data supporting the findings of this study are available upon reasonable request from the corresponding author.
